# MicroRNA-92b represses invasion-metastasis cascade of esophageal squamous cell carcinoma

**DOI:** 10.18632/oncotarget.7747

**Published:** 2016-02-26

**Authors:** Gang Ma, Chao Jing, Lin Li, Furong Huang, Fang Ding, Baona Wang, Dongmei Lin, Aiping Luo, Zhihua Liu

**Affiliations:** ^1^ The State Key Laboratory of Molecular Oncology, Cancer Institute and Hospital, Chinese Academy of Medical Sciences and Peking Union Medical College, Collaborative Innovation Center for Cancer Medicine, Beijing, People's Republic of China; ^2^ Department of Anesthesiology, Cancer Institute and Hospital, Chinese Academy of Medical Sciences and Peking Union Medical College, Beijing, People's Republic of China; ^3^ Department of Pathology, Cancer Institute and Hospital, Chinese Academy of Medical Sciences and Peking Union Medical College, Beijing, People's Republic of China

**Keywords:** esophageal cancer, microRNA-92b, integrin αV, invasion, metastasis

## Abstract

Invasion and metastasis are major contributors to cancer-caused death in patients suffered from esophageal squamous cell carcinoma (ESCC). To explore the microRNAs involved in regulating invasion-metastasis cascade of ESCC, we established two pairs of sublines (30-U/D and 180-U/D) with distinct motility capacity from two ESCC cell lines (KYSE30 and KYSE180). Screening of the differentially expressed microRNAs identified that microRNA-92b-3p (miR-92b) could dramatically inhibit invasion and metastasis of ESCC cells *in vitro* and *in vivo*. Subsequent studies showed that miR-92b exerted its inhibitory function through suppressing the expression of integrin αV (ITGAV), which further reduced phosphrylated FAK and impaired Rac1 activation. Moreover, higher expression of miR-92b in ESCC tissues correlated inversely with lymph node metastasis and indicated better prognosis. Together, these results for the first time describe how miR-92b suppresses the motility of ESCC cells and provide a promise for diagnosis or therapy of ESCC invasion and metastasis.

## INTRODUCTION

Esophageal carcinoma, including esophageal squamous cell carcinoma (ESCC) and esophageal adenocarcinoma (EAC), ranks the sixth lethal cancer among males and the ninth deadly malignance among females around the world. Its overall five-year survival rate is less than 25% mainly due to extensive invasion into lymph nodes and distant metastasis [1–3]. Invasion and metastasis of ESCC cells are a complex process consisting of multiple events [4–6], among which directed migration of tumor cells is critical in nearly all steps of invasion–metastasis cascade [7, 8]. Although previous studies have shed a light on initiation and progression of ESCC [9–11], our understanding of directed motility of ESCC cells in invasion-metastasis cascade still remains poor. In this study, we explored how the directed migration of malignant cells is regulated in invasion and metastasis of ESCC.

A myriad of molecules involve in modulating invasion-metastasis cascade of tumor cells [12]. MicroRNAs, which inhibit gene expression at post-transcriptional level, promote or inhibit cancer cell motility in two ways [13]. One mode is that microRNAs function intracellularly. In breast cancer cells, miR-10b decreased HOXD10 level to enhance metastasis and miR-335 inhibited SOX4 expression to suppress epithelial-mesenchymal transition (EMT) are examples of the first mode [14, 15]. Besides, crosstalk between tumors and microenvironment reveal how microRNAs regulate invasion and metastasis of tumor cells. One piece of evidence is the pro-metastasis of miR-105 in breast cancer. MiR-105 was secreted by breast cancer cells and then transported via exosome into adjacent endothelia cells, where this microRNA reduced ZO-1 expression and undermined the endothelial barrier to prompt intravasion and extravasion [16]. We have previously found that miR-10b inhibited KLF4 expression, the protein which maintained differentiation of esophageal epithelium and whose overexpression promoted inflammation-induced ESCC in mouse models [17, 18], to stimulate migration and invasion of ESCC cells [19].

Here, we identified that microRNA-92b-3p (miR-92b) could significantly inhibit motility of ESCC cells based on an *in vitro* migration model. Moreover, miR-92b correlated negatively with lymph node metastasis and positively with favorable prognosis of ESCC patients. This finding contradicted the previously reported pro-proliferation and pro-invasion function of miR-92b in other tumor models [20, 21]. In addition, we further explored the underlying mechanisms of miR-92b in ESCC invasion and metastasis, and found that integrin αV (ITGAV) was a genuine target of miR-92b. *In vivo* experiments verified that elevated miR-92b or reduced ITGAV suppressed invasion and metastasis of ESCC cells. Mechanistically, overexpression of miR-92b or silence of ITGAV led to decreased phosphrylated focal adhesion kinase (FAK) and reduced activation of Rac1, both of which were essential mediators of cellular motility in ESCC cells. These results demonstrated that miR-92b was a critical regulator of motility and metastasis in ESCC cells.

## RESULTS

### MiR-92b expression differs between ESCC cell subpopulations with distinct motility capacity

**Figure 1:**
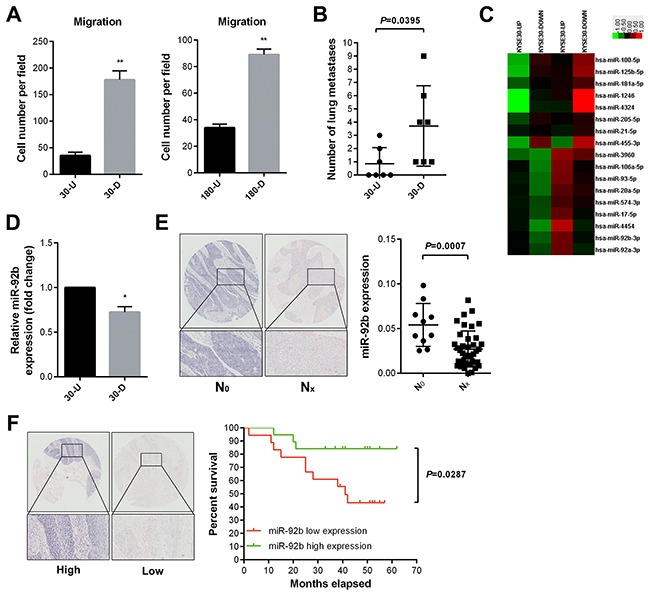
MiR-92b is identified as a negative regulator in ESCC metastasis **A.** More 30/180-D cells penetrated membrane than 30/180-U cells did during 24 hr transwell assay. **B.** Equal amount of 30-D and 30-U cells (5×10^5^ cells) were introduced into immunocompromised mice via tail veins and 30-D cells formed more overt metastases relative to 30-U cells. Data, mean ± SD of seven mice each group. **C.** Differentially expressed microRNAs between 30-U and 30-D subpopulations are shown. **D.** MiR-92b was measured using qPCR, showing that miR-92b expression in 30-U cells was higher than that of 30-D cells. **E.** Representative photographs of ISH results of miR-92b in ESCC specimens (HEso-Squ127lym-01). MiR-92b in ESCC specimens without lymph node metastasis (N_0,_ n = 10) was lower relative to that in those with lymph node metastasis (N_x,_ n = 39). **F.** Representative photographs of ISH results of miR-92b in ESCC specimens (HEso-Squ172Sur-02). Analysis of overall 5-year survival showed that high expression of miR-92b indicated favorable prognosis (Scale bar in E and F, 100 μm).

In order to explore mechanisms modulating ESCC invasion and metastasis, we chose two ESCC cell lines (KYSE30 and KYSE180) for further study. According to two previously published studies [22, 23], we used transwell assay to get two pairs of cell sublines after four rounds of *in vitro* selection, which were named after 30-U/D and 180-U/D respectively. Subsequent study demonstrated that 30/180-D cells possessed stronger capacity of motility than 30/180-U cells *in vitro*, respectively (Figure 1A). Moreover, 30-D cells displayed stronger metastatic capacity than 30-U cells did *in vivo* (Figure 1B).

Next, two independent RNA samples derived from 30-U/D or 180-U/D cells were analyzed using μParaflo^®^Microfluidic Biochip (LC Sciences, Houston, TX, USA). All mature human microRNAs deposited in miRBase (v18) were examined. In total, 17 microRNAs were differentially expressed between 30-U and 30-D cells, among which 9 were upregulated and 8 were downregulated in 30-U cells compared with that of 30-D cells (Figure 1C). Additionally, 2 microRNAs were upregulated whereas 6 microRNAs were downregulated in 180-D cells relative to that of 180-U cells (Supplementary Figure S1A). Among these candidates, miR-92b expression was higher in 30-U cells than that of 30-D cells (Figure 1D), leading us to speculate that this microRNA could suppress motility and even invasion-metastasis cascade of ESCC cells.

### MiR-92b inhibits lymph node metastasis and indicates favorable prognosis of ESCC patients

To test the aforementioned hypothesis, we firstly assessed the expression of miR-92b in an ESCC tissue microarray (HEso-Squ127lym-01, Outdo Biotech) and found that it correlated inversely with lymph node metastasis (Figure 1E). Because lymph node metastasis usually indicates poor prognosis of ESCC [24], we then analyzed miR-92b expression in another ESCC tissue microarray (HEso-Squ172Sur-02, Outdo Biotech, Figure 1F and Supplementary Table S1). Kaplan-Meier survival curve showed that higher miR-92b expression indicated better prognosis (*p* = 0.0287) (Figure 1F and Supplementary Table S1).

### MiR-92b inhibits migration and invasion of ESCC cells *in vitro*

**Figure 2:**
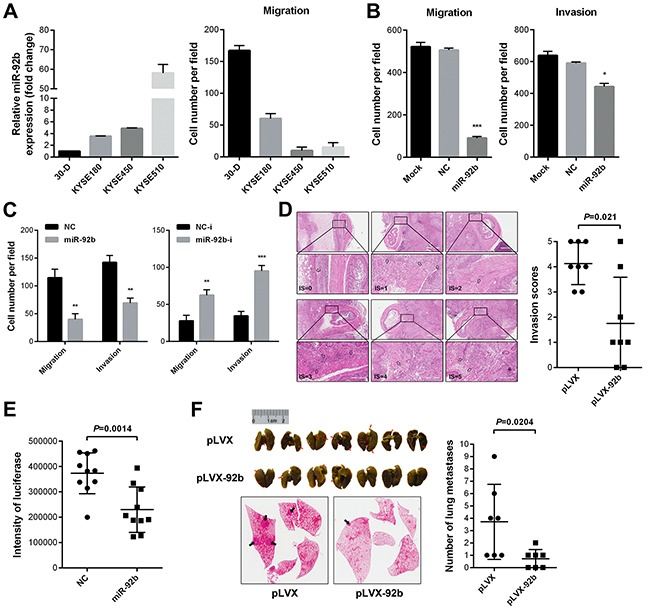
MiR-92b inhibits motility of ESCC cells *in vitro* and *in vivo* **A.** qPCR results showed that miR-92b expression was the highest in KYSE510 cells while the lowest in 30-D cells among a panel of ESCC cell lines. Equal amount of four cell populations (5×10^4^ cells per insert) were incubated in 24-well transwell plate for 24 hr and results showed that 30-D cells manifested strongest motility capacity. **B.** MiR-92b overexpression in 30-D cells impeded penetration of these transfected cells through membrane. **C.** Elevated miR-92b in KYSE450 cells inhibited migration and invasion of these treated cells *in vitro*. Moreover, reduced miR-92b promoted more KYSE510 cells to penetrate transwell membrane. **D.** MiR-92b retarded the process of 30-D cells invading periesophageal muscle (*p* = 0.021). Showed are representative haematoxylin and eosin (H&E) stained specimens of six invasion level (Scale bar in D, 200 μm). Mann-Whitney test was used to compare the difference between the miR-92b and control group. **E.** MiR-92b transfected 30-D cells had weaker pulmonary arrest capacity. The overall bioluminescence intensity, indicating that cells adhere to microvasculature, was calculated by multiplying mean intensity by area. **F.** Photographs of lungs stained with picric acid, which were harvested eight weeks after injection of stably overexpressing miR-92b (pLVX-92b) and control (pLVX) 30-D cells via tail veins (5×10^5^ cells per mice), demonstrate that miR-92b inhibited overt metastases formation in lungs of recipient mice (n = 7).

We asked if miR-92b could suppress motility of ESCC cells *in vitro*. Detection of miR-92b expression in a panel of ESCC cell lines (30-D, KYSE180, KYSE450 and KYSE510) and analyzing motility of the cell lines demonstrated that miR-92b negatively correlated with migration of ESCC cells (Figure 2A). Upon delivery of miR-92b mimic into 30-D cells, the migration and invasion of these recipient cells were dramatically impaired relative to those control cells (Figure 2B, Supplementary Figure S2A and S2B). Additionally, miR-92b retarded the healing velocity of 30-D cells within 24 hours (Supplementary Figure S2C). We also observed that miR-92b, resembling its function in 30-D cells, impeded migration and invasion of KYSE450 cells (Figure 2C, Supplementary Figure S2A and S2D). On the other hand, reduced miR-92b in KYSE510 cells enhanced migration and invasion (Figure 2C, Supplementary Figure S2A and S2E). Taken together, miR-92b could significantly suppress ESCC cell motility *in vitro*.

### MiR-92b suppresses invasion and metastasis of ESCC cells *in vivo*

We used three animal models to assess the effect of miR-92b on invasion-metastasis cascade of ESCC cells. Firstly, we addressed the possibility of miR-92b inhibiting local invasion. ESCC 30-D cells stably overexpressing miR-92b were generated and tested to confirm the inhibitory effect of miR-92b on motility of ESCC cells *in vitro* (Supplementary Figure S3A and S3B). When tumor bulk was appropriate, mice were sacrificed and the subcutaneous masses were obtained, excised, and orthotopically transplanted in the abdominal esophagus. Four weeks after transplantation, we scored the extent of tumor cells invading adjacent periesophageal muscle using haematoxylin and eosin stain (Figure 2D). We found that 2 out of 7 mice implanted with miR-92b tumors were free of invasion (IS0), whereas all mock tumors invaded muscle to different extents (*p* = 0.021, Figure 2D), showing that the control cells manifested more aggressive invasion than the miR-92b- transfected counterparts did.

We then examined whether miR-92b impeded pulmonary arrest of ESCC cells. We introduced miR-92b-transfected and control 30-D cells that were labeled with luciferase into immunocompromised mice via tail veins, respectively. Within 24 hr, we compared lung arrest of the two cell populations. Results showed that fewer miR-92b transfected cells resided in lungs, indicating that miR-92b could undermine attachment of malignant cells to vascular endothelia (*p* = 0.001, Figure 2E and Supplementary Figure S3C). As interactions among transmembrane molecules of circulating tumor cells and endothelia as well as tumor cell size contribute to microvasculature arrest [25], we tested whether miR-92b would diminish bulk of the transfected cells. Flow cytometry did not detect significant alteration in cell volume between the control and the miR-92b-transfected 30-D cells (Supplementary Figure S3D), indicating that miR-92b-induced arrest in capillary beds was largely attributed to changes in transmembrane adhesion molecules.

Lastly, we evaluated whether miR-92b could impair overt metastases formation in lung. Ten weeks after injection, lungs of the recipient mice were harvested. Results showed that metastatic foci in miR-92b-transfected group reduced significantly relative to control group (*p* = 0.02, Figure 2F). However, we failed to observe lung metastases in either control or miR-92b-transfected KYSE450 cells largely due to its poor capacity of forming metastatic nodules *in vivo* (data not shown). These results together indicated that miR-92b could inhibit local invasion and lung colonization of ESCC cells.

Previous studies demonstrated that miR-92b stimulated proliferation of glioma and non-small lung cancer cells [20, 21]. However, miR-92b did not inhibit or promote proliferation of these transfected ESCC cells (Supplementary Figure S4A–S4E). Moreover, neither increased nor decreased expression of miR-92b stimulated apoptosis in ESCC cells (Supplementary Figure S4F–S4I). Collectively, these results demonstrated that miR-92b did not impair cellular viability to suppress ESCC motility.

### ITGAV is the *bona fide* target of miR-92b

In order to understand the inhibitory mechanisms of miR-92b, we analyzed *in silico* the potential targets of miR-92b using seven online algorithms (Supplementary Figure S5A). We chose seven candidates involved in cellular motility or tumor invasion. Luciferase reporter assay excluded the possibility of JMY, SOX4, TOB1, USP28, and WASL as targets of miR-92b (Supplementary Figure S5B).

**Figure 3:**
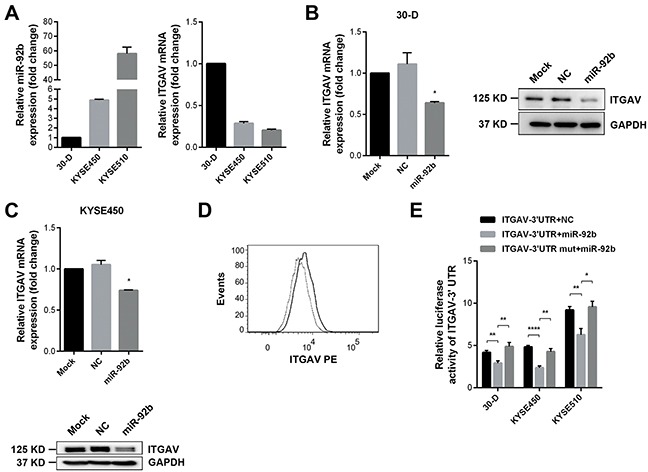
ITGAV is one genuine target of miR-92b **A.** In 30-D, KYSE450 or KYSE510 cells, qPCR results showed that higher miR-92b expression corresponded to lower mRNA level of ITGAV. **B.** and **C.** Immunoblots and qPCR results showed that elevated expression of miR-92b in 30-D (B) and KYSE450 cells (C) reduced ITGAV at both mRNA and protein level. **D.** MiR-92b reduced membrane expression of ITGAV in 30-D cells. Bold line indicates the control cells and dotted line demonstrates the miR-92b-transfected counterparts. **E.** Luciferase activities were assessed 24 hr after co-transfections of pISO-ITGAV-3′UTR or pISO-ITGAV-3′UTR-mut with miR-92b or control oligos in 30-D, KYSE450, and KYSE510 cells respectively, showing that miR-92b directly bound to ITGAV 3′UTR.

We found that miR-92b correlated reversely with the ITGAV expression in 30-D, KYSE450, and KYSE510 cells, indicating that this microRNA probably inhibited expression of ITGAV (Figure 3A). Ensuing studies showed that increased miR-92b in 30-D and KYSE450 cells reduced ITGAV at both mRNA and protein level (Figure 3B and 3C). Also, membrane expression of ITGAV in 30-D cells decreased as the result of miR-92b overexpression (Figure 3D and Supplementary Figure S5C). Moreover, endogenous expression of ITGAV in 30-D, KYSE450, and KYSE510 cells increased significantly upon the transfection of miR-92b inhibitor (Supplementary Figure S5D).

Then, we cloned putative miR-92b binding sites from ITGAV 3′UTR into the reporter plasmid and subsequent dual-luciferase reporter assay demonstrated that miR-92b could reduce luciferase activity significantly in ESCC cells; whereas mutation of the binding sites restored the luciferase activity in these transfected cells (Figure 3E and Supplementary Figure S5E). Additionally, silence of endogenous miR-92b in 30-D, KYSE450, and KYSE510 cells could enhance luciferase activity, further confirming that miR-92b suppressed ITGAV expression by directly binding to 3′UTR of this integrin (Supplementary Figure S5F).

### ITGAV is implicated in invasion-metastasis cascade of ESCC cells

**Figure 4:**
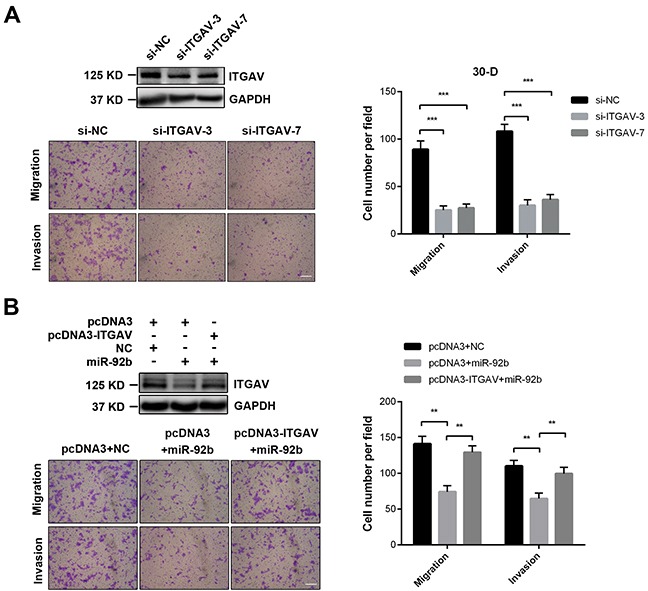
ITGAV stimulates motility of ESCC cells *in vitro* **A.** Specific siRNAs against ITGAV (50 nM) inhibited migration and invasion of 30-D cells *in vitro*, compared with the same cells transfected with control oligos. **B.** ITGAV re-expression promoted more miR-92b-transfected 30-D cells to penetrate membrane relative to these control counterparts. At least three fields were randomly collected. Scale bars in A and B, 400 μm.

As ITGAV was a genuine target of miR-92b, we next tested whether this integrin promoted motility of ESCC cells. Decreased expression of ITGAV debilitated migration and invasion of 30-D cells *in vitro* (Figure 4A). And ITGAV re-expression rescued miR-92b-impaired motility of 30-D cells (Figure 4B). However, ITGAV overexpression in KYSE510 cells failed to significantly promote motility of these transfected cells, which was probably due to deficient expression of genes essential for migration and invasion (such as FAK or Rac1/2/3) (Supplementary Figure S6).

**Figure 5:**
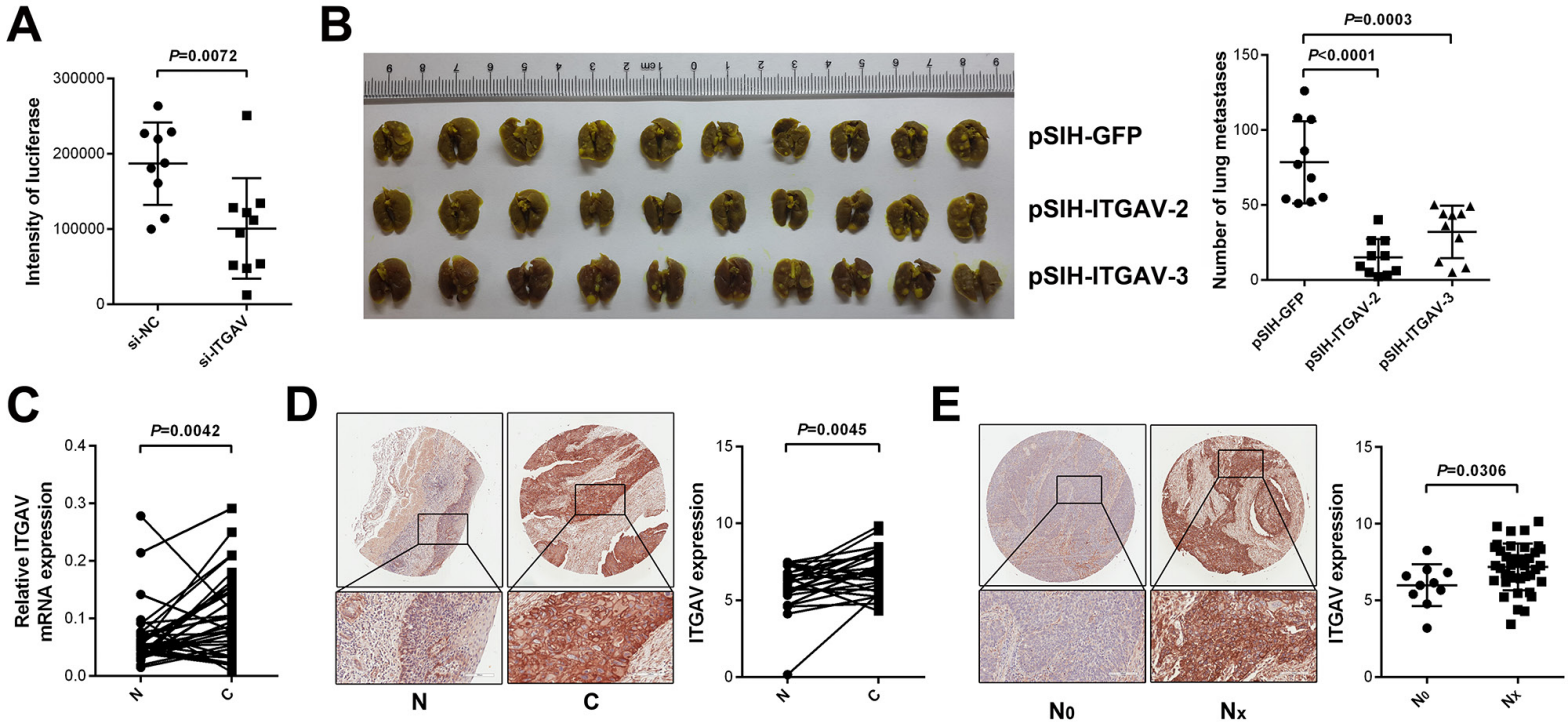
ITGAV promotes metastasis of ESCC cells *in vivo* **A.** ITGAV was responsible for pulmonary arrest of circulating 30-D cells. Data, mean ± SD of overall luciferase intensity. **B.** Decreased ITGAV significantly attenuated macrometastases formation of 30-D cells in experimental lung metastasis model. Pulmonary metastasis in ITGAV experiment was quantified by counting metastatic foci. **C.** ITGAV elevated in ESCC tissues (C) relative to adjacent normal counterparts (N) at mRNA level. GAPDH acted as the internal control of qPCR. **D.** Significantly increased ITGAV was detected in ESCC tissues (C), compared with that of adjacent normal tissues (N) at protein level. **E.** ITGAV expression correlated positively with lymph node metastasis of ESCC. The tissue microarray (HEso-Squ127lym-01) was stained using specific antibody against ITGAV (1:200) and expression of the integrin was analyzed using ImageScope (Scale bars in D and E, 100 μm).

Moreover, we found that silence of ITGAV, resembling miR-92b function in pulmonary arrest, attenuated adhesion of 30-D cells to pulmonary capillaries (*p* = 0.007, Figure 5A and Supplementary Figure S7A), and reduction in ITGAV did not influence cell size either (Supplementary Figure S7B). When calculating metastatic nodules in lungs dissected from the sacrificed immunocompromised mice, we found that ITGAV reduction could undermine colonization of 30-D cells in lung (*p* < 0.001, Figure 5B and Supplementary Figure S7C).

Both quantitative real-time polymerase chain reaction (qPCR) and IHC results revealed elevated ITGAV expression in ESCC relative to adjacent normal tissues (*p* < 0.01, Figure 5C and 5D), which agreed with three published data (GSE20347, GSE23400, and GSE29001, Supplementary Figure S8A); in particular, in two cases higher expression of ITGAV was detected in invasive fronts than that in central regions, which suggested that ITGAV probably stimulated invasion of ESCC cells (Supplementary Figure S8B). Additionally, increased ITGAV correlated positively with lymph node metastasis in ESCC specimens (*p* = 0.002, Figure 5E). *In vivo* assay demonstrated that decreased ITGAV did not significantly retard growth of 30-D cells (Supplementary Figure S9). However, we did not detect the inverse correlation between miR-92b and ITGAV in ESCC specimens when analyzing ISH and IHC results from the same ESCC tissue microarray HEso-Squ127lym-01 (data not shown). This result was probably due to the complex mechanisms other than microRNAs underlying the regulation of ITGAV *in vivo*. Together, we demonstrated that miR-92b inhibited migration and metastasis of ESCC cells through suppressing ITGAV expression.

### MiR-92b impedes TGAV-FAK-Rac1 pathway

**Figure 6:**
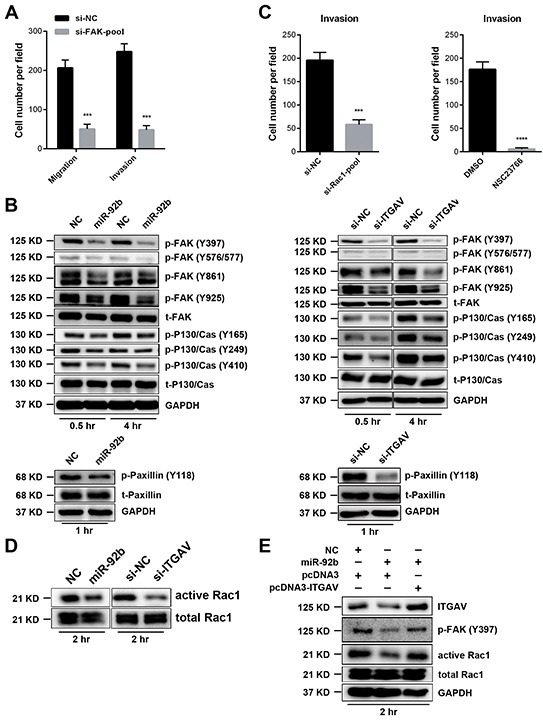
MiR-92b impedes ESCC cell motility by modulating ITGAV-FAK-Rac1 pathway **A.** Knockdown of FAK repressed migration and invasion of 30-D cells *in vitro*. **B.** Under circumstance of chemotaxis, transfection of miR-92b (100 nM) or siRNAs against ITGAV (50 nM) in 30-D cells reduces level of phosphrylated FAK, P130/Cas, and Paxillin at the indicated time points. **C.** Knockdown of Rac1 or inhibition of Rac1 activation using NSC23667 impeded motility of 30-D cells *in vitro*. **D.** Overexpressed miR-92b or reduced ITGAV undermined Rac1 activation. Forty-eight hours after introduction of miR-92b (100 nM) or siRNAs against ITGAV (50 nM) into cells, transfected 30-D cells and control counterparts were harvested under chemotaxis circumstance at 2 hr and the level of activated Rac1 was assessed. **E.** Re-expression of ITGAV could restore the level of p-FAK (Y397) and activated Rac1. These cells were harvested under chemotaxis condition at 2 hr and the level of phosphrylated FAK (Y397) and active Rac1 were analyzed.

After validating functions of ITGAV in ESCC cell motility, we wanted to delineate signaling pathways affected by miR-92b. To this end, we firstly focused on FAK because integrins usually relay essential motility signals through this critical kinase [26]. To begin with, silence of FAK undermined migration and invasion of 30-D cells *in vitro* (Figure 6A, Supplementary Figure S10A and S10B). Thereafter, we detected the level of phosphorylated FAK and P130/Cas at indicated time points, showing that elevated miR-92b in 30-D cells decreased the abundance of p-FAK (Y397, Y576/577, Y861 and Y925) and p-P130/Cas (Y165, Y249, and Y410) (Figure 6B). Paxillin phosphorylation is a critical event during focal adhesion formation; in our system, we also observed decreased level of p-Paxillin (Y118) in miR-92b-transfected cells (Figure 6B). On the other hand, inhibition of miR-92b in KYSE510 cells resulted in increased level of p-FAK (Y397) (Supplementary Figure S10C). Reduction in ITGAV, similar to elevated miR-92b, reduced the level of p-FAK, p-P130/Cas and p-Paxillin relative to that of those control cells (Figure 6B).

We then presumed that Rac1 was an essential effector influenced by miR-92b since Rac1 is a key downstream target concerning motility of FAK- P130/Cas pathway [26]. As expected, both silence of Rac1 and pharmaceutical inhibition using NSC23667 could significantly inhibit invasion of 30-D cells (Figure 6C, Supplementary Figure S10D and S10E). Besides, we found that miR-92b or ITGAV depletion reduced the amount of activated Rac1 without influencing activation of total Rho and Cdc42 (Figure 6D and Supplementary Figure S10F). Moreover, re-expression of ITGAV could rescue Rac1 activation and increase the level of p-FAK (Y397) in 30-D cells pre-transfected miR-92b mimics (Figure 6E). Accumulating evidence has corroborated that integrin αvβ5 and αvβ3 were involved in tumor metastasis [27, 28], we therefore detected the membrane expression of these two integrin heterodimers in 30-D cells, showing negative expression of αvβ3 but dramatically decreased αvβ5 responding to elevated miR-92b (Supplementary Figure S11A). Further knockdown of ITGB5 attenuated motility of 30-D cells, partially demonstrating that integrin αvβ5 was probably important in invasion-metastasis cascade of ESCC (Supplementary Figure S11B). Collectively, miR-92b attenuated motility of ESCC cells by suppressing ITGAV–FAK–Rac1 pathway.

## DISCUSSION

MiR-92b, which is a member of miR-25 family, is involved in development of central neural system and progression of several kinds of tumors [29, 30]. Previous reports from glioblastoma and non-small cell lung cancer indicated that miR-92b promoted growth and metastasis of neoplastic cells; whereas absence ofmiR-92b contributed to transformation of normal human B lymphocytes through epigenetic mechanisms [31]. In ESCC, miR-92b did not suppress proliferation or stimulate apoptosis, but it hampered motility of malignant cells. These results agree with the doctrine that one specific microRNA could play various (sometimes even opposite) roles in distinct genetic contexts [32].

Integrins consists of 18 α and 8 β members, which form 24 heterodimers to crosslink ECM and cytoskeleton [33]. Accumulating evidence has established that formation of overt metastases resulted from successful adaption of malignant cells to constantly changing microenvironments. Since they regulate cellular adhesion, motility and survival, integrins undoubtedly assist cells disseminated from primary lesions during each step of invasion-metastasis cascade [34]. Previous study demonstrated that integrin αVβ3 in melanoma cells would stably adhere to E-selectin of endothelial cells to stimulate ensuing transmigration [35]. Moreover, ITGAV promoted metastasis in several types of tumors such as breast cancer and prostate cancer [36, 37]. In this study, miR-92b in murine models of pulmonary metastasis impaired lung retention of circulating cancer cells in the short term and impeded overt metastases formation in the long term; similar results could be observed in ITGAV-silenced 30-D cells. In our study, integrin αVβ5, not αVβ3 was detected in 30-D cells and membrane expression of integrin αVβ5 dropped dramatically upon transfection of miR-92b mimic. However, whether this is the only integrin heterodimer which functions in 30-D cells still needs further investigation.

Responding to variant stimulus integrins activate or suppress different signaling pathways, among which FAK is one critical signal hub [26]. We found that miR-92b decreased the level of p-FAKs and relevant scaffold proteins such as p-paxillin and p-p130/Cas. Furthermore, elevated miR-92b or ITGAV depletion in 30-D cells diminished activated Rac1 that is essential for ESCC motility. The preliminary data, however, raised more questions about the precise roles of ITGAV in ESCC pathology. Signals are relayed usually through β integrins recruiting and binding to other signaling scaffold proteins. We found that knockdown of ITGB1 or ITGB5 retarded motility of 30-D cells respectively. Moreover, ITGB1 depletion also reduced p-FAKs but failed to cause pulmonary arrest (data not shown). Thisshowed that functions of these integrins overlapped under diverse conditions and more efforts are needed to delineate complicated mechanisms of individual integrin.

In summary, we used *in vitro* and *in vivo* models to reveal that miR-92b suppressed invasion-metastasis cascade of ESCC cells by inhibiting ITGAV-FAK-Rac1 pathway, providing a promise in diagnosis and treatment of ESCC. Notably, transwell assay, although it is widely used in assessing migration and invasion of targeted cells, is biased since design of the inserts is prone to highlight roles of integrins [38]. Additionally, genetic backgrounds vary greatly among ESCC cell lines, the differentially expressed microRNAs and mRNAs (GSE67510) were rather different between 30-U/D and 180-U/D cells. Accordingly, this investigation has just touched a tip of the iceberg and delicate *in vivo* models are required to fully understand invasion and metastasis of ESCC.

## MATERIALS AND METHODS

### Cell culture and reagents

ESCC cell lines (KYSE30/180/450/510), which were generous gifts from Dr. Y. Shimada (Kyoto University, Kyoto, Japan) [39], and derived sublines (30-U/D and 180-U/D) were cultured in RPMI1640 with 10% FBS, ampicillin (100 U/ml), and kanamycin (100 mg/ml). All ESCC cells were authorized by STR analysis. HEK293T cells were obtained from ATCC (Manassas, VA, USA) and cultured in DMEM with 10% FBS, ampicillin (100 U/ml), and kanamycin (100 mg/ml). All cells were maintained under humanized condition (37°C, 5% CO_2_) and continual culture duration did not exceed two months.

Two paraffin-embedded ESCC tissue microarrays were purchased from Shanghai Outdo Biotech Co., Itd (Shanghai, China), of which HEso-Squ127lym-01 was for detecting the role of molecules in ESCC lymph node metastasis and HEso-Squ172Sur-02 was for the role of miR-92b in overall survival of ESCC patients,. The experiments on tissue specimens were approved by the ethical committee of the Chinese Academy of Medical Sciences Cancer Institute.

Synthesized miR-92b, inhibitor against miR-92b, and negative control oligos were purchased from Thermo Fisher Scientific (Waltham, MA, USA). NSC23766 was commercially sourced from Tocris Bioscience (Bristol, United Kingdom) and used during transwell assay as previously described [40].

### Isolation of ESCC cell subpopulations

We used 6-well transwell plates containing inserts (Corning, One Riverfront Plaza, NY, USA) to set up an *in vitro* migration model to obtain ESCC cell sublines (Supplementary Figure S1B). In brief, KYSE30 and KYSE180 cells (70-80% confluent) were serum starved for 24 hr before they were digested and suspended in RPMI1640 without FBS. Cell density was adjusted to 5×10^5^ cells/ml and 1 ml cell suspension was added into one insert pre-incubated with 0.5 ml RPMI1640 without FBS. The lower chamber was added with 2.5 ml RPMI1640 with 20% FBS. After 24 hr incubation under circumstance of 37°C and 5% CO_2_, cells from upside (U) and downside (D) of the membrane were harvested and expanded, respectively. In the ensued three rounds of selection, only the upside cells derived from the first-generation of U subpopulation and the membrane-penetrated cells from the original D subline were obtained. After four rounds of continual screening, we got two pairs of ESCC cell sublines, which were denoted as 30-U/D and 180-U/D respectively.

### Plasmids and lentivirus

The expression plasmid pcDNA3-ITGAV-EGFP was kindly provided by Dr. H. Q. Zhang (Peking University, Beijing, China). We subcloned ITGAV into pcDNA3-myc and the *myc* tag was attached to C terminal of these two integrins. Potential binding sites of miR-92b in 3′UTR of the predicted genes were cloned into pISO, which was a gift from Dr. D. P. Bartel of Massachusetts Institute of Technology (Cambridge, MA, USA). We extracted genome DNA from KYSE30 cells and cloned sequence containing miR-92b into pLVX-IRES-Neo. We designed at least three candidate shRNAs for ITGAV using *RNAi Central* developed by G. J. Hannon *et al*. (Cold Spring Harbor Laboratory, USA). Designed oligos were synthesized, annealed and constructed into pSIH-puro according to instructions of the plasmid vendor SBI (San Francisco, CA, USA). All constructions were verified by sequencing in SinoGenoMax (Beijing, China). The primers and shRNA oligos are listed in Supplementary Table S2.

Lentivirus preparation was based on a protocol shared by G. Tiscornia*et al* [41]. In brief, HEK293T cells were maintained around 50-60% confluence in 10 cm culture dishes when transfected with plasmids. As for pLVX-IRES-Neo system, we mixed 7.5 μg of expression plasmid with 12.5 μg of package plasmids while as for pSIH-puro system 5 μg of knockdown plasmid with 15 μg of package plasmids were mixed. Sixty μl of Lipofectamine 2000 per dish (Invitrogen) was added into Opti-MEM containing plasmids mixture. Gently mixed, incubated for 10 min under room temperature, the mixtures of plasmids and transfection reagent were then dropped randomly into cultured HEK293T cells. When HEK293T cells grew to 80-90% confluence, fresh DMEM with 10% FBS was added in culture dishes and after around 24 hr the culture medium was collected, centrifuged (4.5×10^3^ g, 4°C), filtered (0.45 μm pores) and stored in −80°C refrigerator in aliquots.

### Screening of differentially expressed microRNAs

After four rounds of selection, we expanded obtained ESCC 30-U/D and 180-U/D subpopulations. We seeded these expanded cells, which had at most 3 passages, in 10 cm culture dishes at a proper density and harvested these cells using 4 ml Trizol per dish when these cells reached around 80% confluence within 24 hr. Two independent pairs of 30-U/D and 180-U/D samples were obtained and analyzed using Agilent 2100 Bioanalyzer to ensure integrity of RNA. Qualified RNA samples were then subjected to microRNA array analysis using μParaflo^®^Microfluidic Biochip covering all human microRNAs of miRBase v18 (LC Sciences, Houston, TX, USA). Raw data was processed by experts from LC Sciences before sending back to us. Raw data of the array experiment were submitted to Gene Expression Omnibus under GSE67510.

### Transfection and transduction

The untransfected cells, cells transfected with negative control oligos, miR-92b mimic or specific miR-92b inhibitor are labeled as MOCK, NC/NC-i, miR-92b, or miR-92b-i, respectively. ESCC cells transfected with siRNAs against ITGAV or negative control oligos were labeled as si-ITGAV or si-NC, respectively. Since rescue experiments required co-transfection of microRNA and plasmids, we adopted two-step transfection method, which included transfection of plasmids in the first phase and then introduction of miR-92b mimic and negative control oligos. The procedure was completed within five hours since onset of transfection. During transfection, RPMI1640 or DMEM without ampicillin and kanamycin was used and after 18-24 hours fresh culture medium was added in place of the transfected medium.

Thawed lentivirus was added into each well of 6-well plate with polybrene (6 μg/ml). Fresh RPMI1640 with FBS was added up to 2 ml per well. Twenty-four hours after addition of lentivirus, culture medium was removed. Infected cells were washed twice with pre-warmed PBS, and fresh RPMI1640 with FBS and G418 (400 μg/ml for 30-D cells or 200 μg/ml for the other KYSE cell lines) or puromycin (1 μg/ml for 30-D cells) was added to get clones. Stable 30-D clones expressing miR-92b or not was labeled as pLVX-92b and pLVX, respectively. Moreover, 30-D cells with stably reduced expression of ITGAV were labeled as pSIH-ITGAV and pSIH-GFP was used as negative control.

### Flow cytometry

We adopted forward scatter (FSC), which is a parameter to measure volume of cells going through flow cytometer detectors, to evaluate whether microRNA or siRNA would alter the size of the transfected cells relative to the control counterparts. In brief, transfected cells were digested gently to single cells 48 hr after delivery of oligos and then subjected to flow cytometry analysis. To detect cell cycle, we used 75% ethanol to fix the harvested cells and stored them in 4°C refrigerator. Before analysis, cells suspended in 400 μl PBS were incubated with 50 μl propidium iodide (1 μg/μl) and 0.5 μl RNase (100 μg/μl) for 30 min in the dark. Apoptosis assay was performed in accordance with FITC-AnnexinV Apoptosis Detection kit instruction of manufacturer (BD, Franklin Lakes, NJ, USA). To detect membrane expression of integrins, we stained the harvested cells with fluorochrome-conjugated antibodies against target molecules (10 μl/ 1×10^6^ cells). The unstained cells and cells stained with corresponding isotype antibodies served as control. Antibodies used in flow cytometry are listed in Supplementary Table S3. All experiments were performed on the same LSR II (BD).

### qPCR and immunoblots

Total RNA from cultured cells or frozen ESCC tissues was extracted according to standard Trizol method (Invitrogen). Five hundred nanograms of RNA were reversely transcribed to complementary DNA (cDNA) in 10 μl reaction system (37°C, 60 min) using Quantscript RT kit produced by Tiangen (Beijing, China). MiR-92b was reversely transcribed using gene specific primer while mRNAs and U6 were converted using random hexamers. Expression level analysis was performed using SYBR Premix Ex Taq^TM^ II (TaKaRa, Japan) on Step-one plus real-time PCR system (Applied Biosystems, Foster City, CA, USA). The 20 μl PCR mixtures in 8-strip tubes were incubated at 95°C for 5 min and then cycling for 40 times between 95°C for 5 sec and 60°C for 30 sec. ROX reference dye was added into reaction mixtures as internal control. Data was normalized to the expression level of GAPDH for mRNAs or U6 for microRNAs and 2^−ΔΔCt^ method was used to evaluate the relative abundance of genes.

Cells prepared for immunoblotting assay were harvested under adherent or chemotaxis condition. As for chemotaxis circumstance, ESCC cells from control and transfected groups were suspended in FBS-free RPMI1640 (5×10^5^ cells/ml) and 1 ml cell suspension was added into one insert of 6-well transwell dishes (Corning, Lowell, MA, USA). In the lower chambers were added RPMI1640 with 20% FBS. Cells, maintained in incubator, were harvested at the indicated time points. Ensuing western blots were carried out according to standard procedure. Briefly, cells were collected in lysis buffer (10 mM Tris-HCl, 150 mM NaCl, 5 mM EDTA, 1% Triton X-100, and 0.25% sodium deoxycholate, pH=7.4) containing protease and phosphatase inhibitors (Roche) and stored in −80°C refrigerator overnight. Frozen cells were thawed on ice and centrifuged (15,000 g, 4°C) for 30 min to remove cellular debris. Then protein concentration was determined using BCA method (Thermo Fisher Scientific) and 20-30 μg protein was boiled for 5 min, separated, transferred to PVDF membranes, blocked with 5% non-fat milk and probed using primary antibodies. Horseradish-peroxidase-conjugated secondary antibodies (1:8000) and substrate (Thermo) were used to detect protein abundance according to instructions. All immunoblots images were acquired by ImageQuant LAS-4000 System (GE).

### Transwell and wound healing assay

*In vitro* migration or invasion assay, in which inserts were pre-coated with 3.6 μl Matrigel (BD) in 100 μl RPMI1640 without FBS, was performed as previously described [19]. Images of at least three randomly selected fields were acquired using Nikon Biophot (Japan) equipped with camera and software LAS v4.6. The penetrated cells were counted with the help of Adobe Photoshop CS4 and data were represented as mean ± SD.

Wound healing assay was used to confirm the result of transwell experiment. Generally, after 36-48 hr transfection, equal amount of miR-92b-treated and control 30-D cells (5×10^5^ cells per well) were added into 12-well culture dish. When cells grew nearly to 100% confluence, scratches were made using 10 μl pipette tips and culture medium was replaced with fresh RPMI1640 with 10% FBS. Images of gap sizes from several randomly fields were acquired at the beginning (0 hr) and the endpoint (24 hr) of experiment using Leica DM 1L LED inverted microscope (German) equipped with camera and software LAS 4.3.

### Dual-luciferase reporter assay

The wildtype biding site of miR-92b in 3′UTR of ITGAV was cloned using Phusion High-Fidelity DNA Polymerase (NEB, Ipswich, MA, USA) into the downstream of firefly luciferase cassette in pISO. The mutations in binding sites were constructed using KOD-Plus-Mutagenesis Kit (TOYOBO, Japan) based on polymerase chain reaction. The mutation primers are listed in Supplementary Table S2. The pISO plasmid containing wild type or mutated miR-92b recognized sites was co-transfected with a Renilla luciferase vector and miR-92b or negative control oligos into 30-D, KYSE450, or KYSE510 cells, which were maintained in 96-well plates, in quadruplicate respectively. After 24 hr, luciferase activities were assessed using Dual-Luciferase Reporter Assay (Promega, Madison, WI, USA) from four parallel wells.

### Detection of active small GTPase

ESCC cells from the control or transfected (miR-92b expression or ITGAV knockdown) group were harvested under chemotaxis condition after 2 hr incubation. Subsequent procedure followed the instruction of Cell Signaling Technology (Danvers, MA, USA).

### Animal experiments

*In vivo* growth assay was performed by injecting 1×10^6^cells subcutaneously and after four weeks tumors were removed and weighed to evaluate whether proliferation of cells were affected. The control and treated ESCC cells were injected in the same immunocompromised mice to minimize variations among individual animal.

Local invasion model of ESCC cells was based on a report from S. J. Gros *et al* (Supplementary Figure S1C) [42]. One million pLVX or pLVX-92b cells suspended in PBS were injected subcutaneously. When grew to about 0.8 cm^3^, tumors were removed from the anaesthetized mice (2.4% tribromoethanol) and chopped to around 1 mm^3^ fragments, which were then implanted to abdominal esophagus of anaesthetized mice (eight mice per group). After four weeks, the implanted tumors with esophagus were got from sacrificed mice, sectioned randomly, and analyzed using haematoxylin and eosin stain. A senior pathologist (Dr. D. M. Lin) with us evaluated the invasion extent of ESCC cells into periesophageal muscle. Based on invasion extent, we used “Invasion Score” (IS) to statistically compare invasion capacity of pLVX cells with that of these pLVX-92b cells. See the Supplementary Table S4 for details of IS0-IS5.

We delivered microRNAs or siRNAs and negative control oligos respectively into 30-D cells stably expressing luciferase and injected 5×10^5^ cells in 100 μl PBS into tail veins of mice. Within 24 hr, we injected D-luciferin (Caliper Life Sciences, Hopkinton, MA, USA) diluted in DPBS (150 μg/ml) into abdomen of each mice (10 μl D-luciferin /g mouse body weight). After 5 min, mice injected with luciferin were anaesthetized using 2.4% tribromoethanol (100 μl per 10 g mouse body weight). Images were acquired using Photometrics I.C.E. Chemi & Fluo System P80 (Tucson, AZ, USA) and the exposure time was set to 8 min unless specifically stated. Ensuing analysis of images was performed by using Slidebook 4 developed by Intelligent Imaging Innovations, Inc. (Denver, CO, USA) to multiply mean intensity of bioluminescence by area. Moreover, in order to detect lung colonization of ESCC cells, we also introduced the manipulated and control 30-D cells (5×10^5^ cells in 100 μl PBS) into immunocompromised mice through tail veins, respectively. Lungs were obtained around 10-12 weeks after injection, fixed with 4% paraformaldehyde, and then stained using 3% picric acid to count metastatic foci or weighed.

### *In situ* hybridization and immunohistochemistry

As for detection of miR-92b *in situ*, specimens were incubated with proteinase K (15 μg/ml) under 37°C for 10 min. We then washed specimens with PBS and dehydrated using sequentially increased concentrations of ethanol (70%, 96% and 100%). MiR-92b probe (40 nM) was added on specimens and ensuing incubation was performed under 60°C for 1 hr. When incubation ends, we washed specimens briefly in pre-warmed 5×, 1× and 0.2× SSC (60°C) in sequence. The primary antibody against DIG (1:800, Roche) was incubated with specimens under room temperature for 60 min and the substrate NBT/BCIP (Roche) was added and incubated in dark for about 15 min. When specific blue signal was observed and KTBT was used to stop further reaction. All procedures were performed under RNase-free condition. Further analysis was completed by using Image-Pro Plus (Media Cybermetics, Rockville, MD, USA). When exploring the relationship between miR-92b and prognosis, we categorized 39 ESCC patients into High and Low group based on the median expression level of miR-92b (median level is 3.098182).

The paraffin-embedded ESCC tissue chip (HEso-Squ127lym-01) was stained with primary antibody against ITGAV (1:200) (Supplementary Table S3) and further visualization was performed using Vectastain ABC kit and DAB chromagen (Vector Laboratories, Burlingame, CA, USA). Mounted specimens from immunohistochemistry were analyzed by ImageScope system (GE) as described previously [43].

### Statistical analysis

Investigators were not blinded in analyzing data from most experiments other than ISH and IHC assay. Exclusion criteria for ISH and IHC experiments were pre-established. As for animal experiments, difference in *n* of each experiments resulted from factors unrelated to experiments such as death caused by pulmonary embolism. Data were analyzed using two-tailed or paired *Student's t*-test unless stated particularly. All experiments other than histological and animal assays were repeated at least twice. In bar graphs, *, **, and *** indicate *p* < 0.05, 0.01 and 0.001, respectively.

## SUPPLEMENTARY FIGURES AND TABLES


