# Supplementary material for: MicroRNA-92b represses invasion-metastasis cascade of esophageal squamous cell carcinoma
Source: Oncotarget. 2016 Feb 26;7(15):20209–22. doi: 10.18632/oncotarget.7747 (PMC4991448; doi:10.18632/oncotarget.7747)
Supplement: Supplementary file 1 [file oncotarget-07-20209-s001.pdf]

SUPPLEMENTARY TABLES AND FIGURES

Supplementary Table S1: miR-92b Expression and Clinicopathological Features of ESCC

Clinicopathological Features	Cases	miR-92b (%)		*P*-value
		Low	High	
Gender				
Male	26	14 (53.8)	12 (46.2)	0.728
Female	11	5 (45.5)	6 (54.5)	
Age				
≤60	17	10 (58.8)	7 (41.2)	0.515
>60	20	9 (45)	11 (55)	
Tumor size (cm)				
≤4	20	8 (40)	12 (60)	0.191
>4	17	11 (64.7)	6 (35.3)	
Histological grade				
I	18	9 (50)	9 (50)	1.000
II-III	19	10 (52.6)	9 (47.4)	

NOTE: The result was analyzed by the Fisher's Exact Test.

Supplementary Table S2: List of primers and oligos used in this investigation

Application	Sequence
**Clones**	
miR-92b	F: TTCTCGAGTTCTGGGACTCCGCAAACTC
	R: TTTCTAGATTACTCAGGCACTAACCCCA
ITGAV 3'UTR	F: TTGAGCTCCTAGGATTATTAATAAAAGCCACAT
	R: TTGCTAGCAAATAACCTCACTTAATCACTCT
JMY 3'UTR	F: TTGAGCTCTCATTCAAGATTGGTTCTGAT
	R: TTGCTAGCACAGTAGATCTCTGTCAACCC
TOB1 3'UTR	F: TTGAGCTCCCATCTTAAAGACATCAACTA
	R: TTGCTAGCTAAACTATTTCAGTCCCTCTT
SOX4 3'UTR	F: TTGAGCTCACAGTATCCCTTAACCTGCCA
	R: TTGCTAGCTTCCAGCTGATTTTATTTCCT
USP28 3'UTR	F: TTGAGCTCGAAGAGCATTGTGTAACTTGA
	R: TTGCTAGCAAACAAATACTTTTATTGCACAT
WASL 3'UTR	F: TTGAGCTCGGTGAAATACTAAACACTACT
	R: TTGCTAGCTTTTGTCAGTCTCACATATAG
**microRNA reverse transcription**	
miR-92b	CTCAACTGGTGTCGTGGAGTCGGCAATTCAGTTGAGG GAGGCCG
**qPCR detection**	
miR-92b	F: ACACTCCAGCTGGGTATTGCACTCGTCCCG
	R: TGGTGTCGTGGAGTCG
ITGAV	F: TTGTTGCTACTGGCTGTTTTG
	R: TCCCTTTCTTGTTCTTCTTGAG
GAPDH	F: TGCACCACCAACTGCTTAGC
	R: GGCATGGACTGTGGTCATGAG
U6	F: CTCGCTTCGGCAGCACA
	R: AACGCTTCACGAATTTGCGT
**Mutations**	
ITGAV UTR mut-1	F: GATCCGTTATGCTTATATTTCATTTTAATT
	R: TGCTGCATATTTCTATCGTTCAAAC
ITGAV UTR mut-2	F: ACTAGCATTAACAATGTAACCAAATCTAGATA
	R: TTGAGAACAATGACAAAAAACATC
**shRNA oligos**	
ITGAV sh-2 target	GCGGGACCATCTCATCACTAAG
ITGAV sh-3 target	ATCTCATAATTTCATGCACAAG
GFP shRNA target	CGAGAAGCGCGATCACATG

Supplementary Table S3: Primary antibodies used in this investigation

Primary antibody	catalog	Vendor	Application
ITGAV	3919-1	Epitomics	WB
GAPDH	G8795	Sigma	WB
PCNA	sc-56	Santa Cruz	WB
Caspase-3	sc-56052	Santa Cruz	WB
PARP	sc-23461	Santa Cruz	WB
FAK Antibody Sample Kit	9330	Cell Signaling	WB
Rho-GTPase Antibody Sample Kit	9968	Cell Signaling	WB
Actin Nucleation Antibody Sample Kit	8355	Cell Signaling	WB
Actin Reorganization Antibody Sample Kit	9967	Cell Signaling	WB
Paxillin	2542	Cell Signaling	WB
Phospho-Paxillin (Tyr118)	2541	Cell Signaling	WB
Phospho-Akt (Thr308)	2965	Cell Signaling	WB
Akt (pan)	4685	Cell Signaling	WB
p130 Cas	13383	Cell Signaling	WB
Phospho-p130 Cas (Tyr165)	4015	Cell Signaling	WB
Phospho-p130 Cas (Tyr249)	4014	Cell Signaling	WB
Phospho- p130 Cas (Tyr410)	4011	Cell Signaling	WB
Phospho-FAK (Tyr861)	ab4804	Abcam	WB
Sheep anti-DIG-AP	1109327910	Roche	ISH
ITGAV	ab179475	Abcam	IHC
ITGAV	FAB1219P	R&D	FC
Integrin αVβ3	FAB3050P	R&D	FC
Integrin αVβ5	FAB2528P	R&D	FC
Isotype rat IgG_{2a}, κ	555843	BD	FC
Isotype mouse IgG_1	IC002P	R&D	FC

Supplementary Table S4: Description of *invasion scores* (IS) used in local invasion model

Invasion Score Category	Description
IS = 0	No attachment to muscle tissues
IS = 1	Evident adhesion to muscle tissues but no invasion
IS = 2	Invasion of malignant cells into longitudinal muscle
IS = 3	Invasion of malignant cells into circular muscle
IS = 4	Break of the muscle but no invasion into submucosa
IS = 5	Invasion into submucosa and mucosa

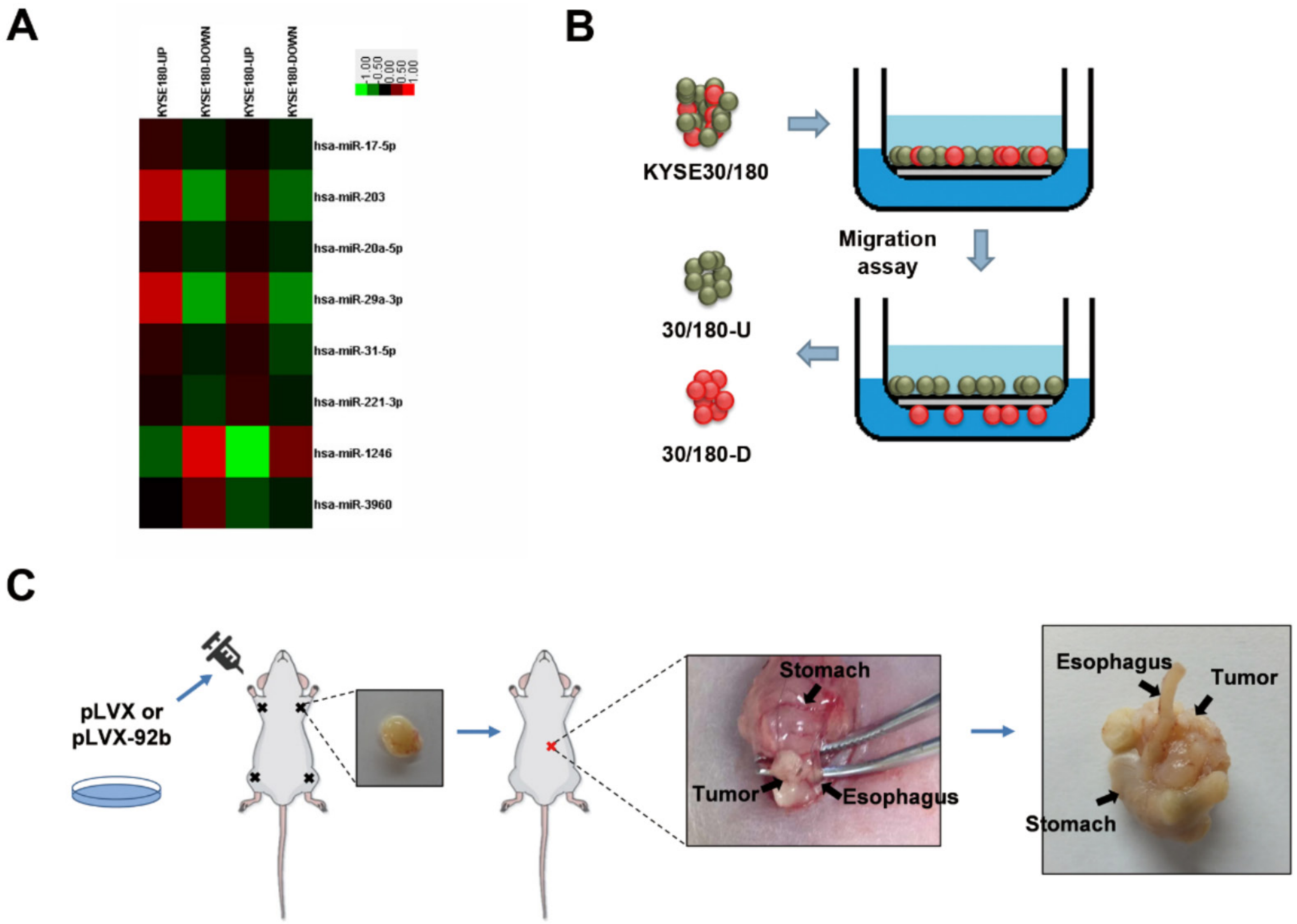

Supplementary Figure S1: A. Two pairs of independent RNA samples derived from 180-U (KYSE180_UP) and 180-D (KYSE180_DOWN) sublines were collected and subjected to microRNA expression screens. The differentially expressed microRNAs (change fold > 2) are showed in the heat map. **B.** Illustration of ESCC subpopulation isolation based on *in vitro* transwell assay shows that after four continual screens 30/180-D sublines with stronger motility capacity relative to 30/180-U were selected. **C.** Illustration of establishment of ESCC local invasion model. Equal amount of 30-D cells with stable miR-92b expression or control counterparts wereinjected subcutaneously on immunocompromised mice (1×10^6 cells per injection). When tumors grew to around 0.8 cm^3, they were harvested, chopped, and transplanted to esophagus of the recipient mice. One month after the transplantation, tumors with adjacent stomach and esophagus were obtained and invasion extend was analyzed.

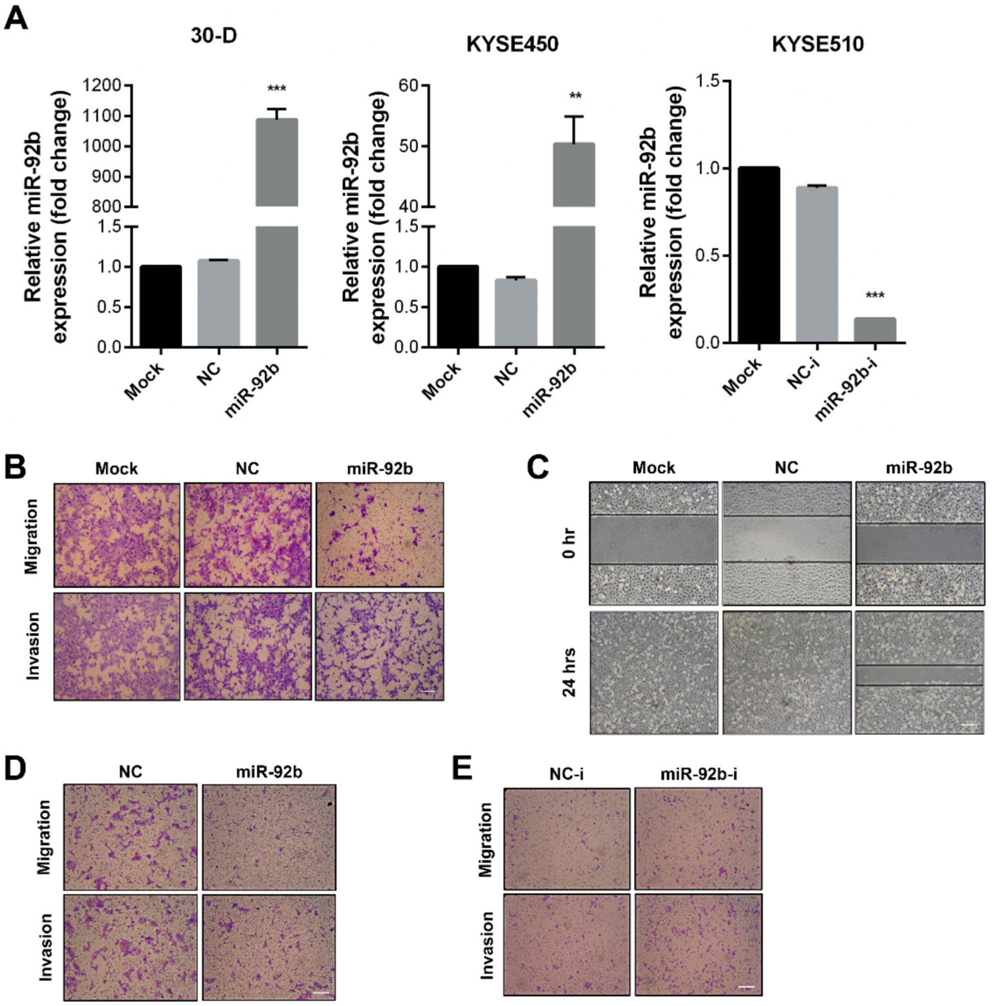

Supplementary Figure S2: MiR-92b inhibited motility of ESCC cells *in vitro*. A. qPCR results of enforced and reduced expression of miR-92b in 30-D, KYSE450 and KYSE510 cells. The level of miR-92b in the transfected 30-D and KYSE450 cells with miR-92b mimic were analyzed 36-48 hr after transfection and in KYSE510 cells were detected around 96 hr after two ensuing rounds of transfections of miR-92b inhibitor. **B.** and **C.** Increased expression of miR-92b made less 30-D cells relative to control counterparts to penetrate membrane in transwell assays (B). Moreover, representative photographs of gap at the beginning (0 hr) and the endpoint (24 hr) of wound healing assay confirmed the inhibitory effect of this microRNA on motility of 30-D cells (C). Both assays were performed 36-48 hr after delivery of miR-92b into 30-D cells. **D.** and **E.** Increased expression of miR-92b in KYSE450 cells suppressed migration and invasion of these transfected cells (D) whereas decreased level of miR-92b by miR-92b inhibitor (miR-92b-i) promoted KYSE510 cells to penetrate membrane (E). Scale bars, 400 µm.

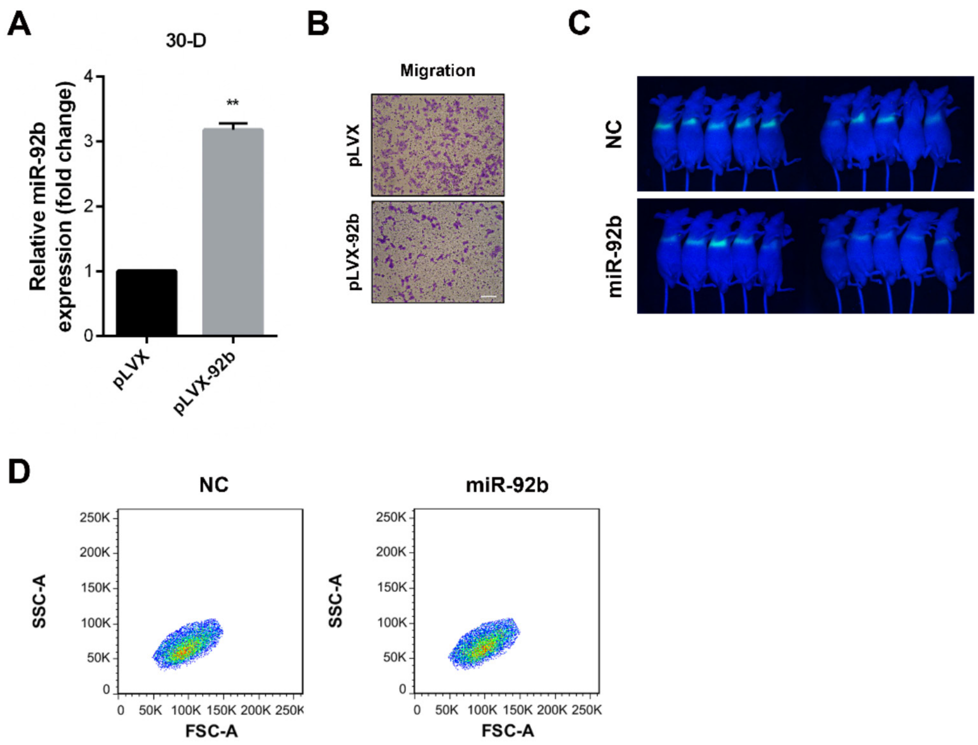

Supplementary Figure S3: MiR-92b impeded invasion-metastasis cascade of ESCC cells *in vivo*. A. and **B.** Stable elevation of miR-92b was engineered in 30-D cells using lentivirus-based system (A) and these cells manifested weaker motility capacity relative to control counterparts as shown by transwell assay (B, Scale bars, 400 µm). **C.** Images show that increased level of miR-92b attenuated adhesion of 30-D cells to microvasculature in lungs. ESCC 30-D cells stably expressing luciferase were transfected with miR-92b and control mimic respectively for 48 hr before they were injected via tail veins. Detection were performed within 24 hr. **D.** Flow cytometry results showed that increased expression of miR-92b exerted little influence on cell volume of 30-D cells based on comparison of forward scatter (FSC) between the control and transfected group.

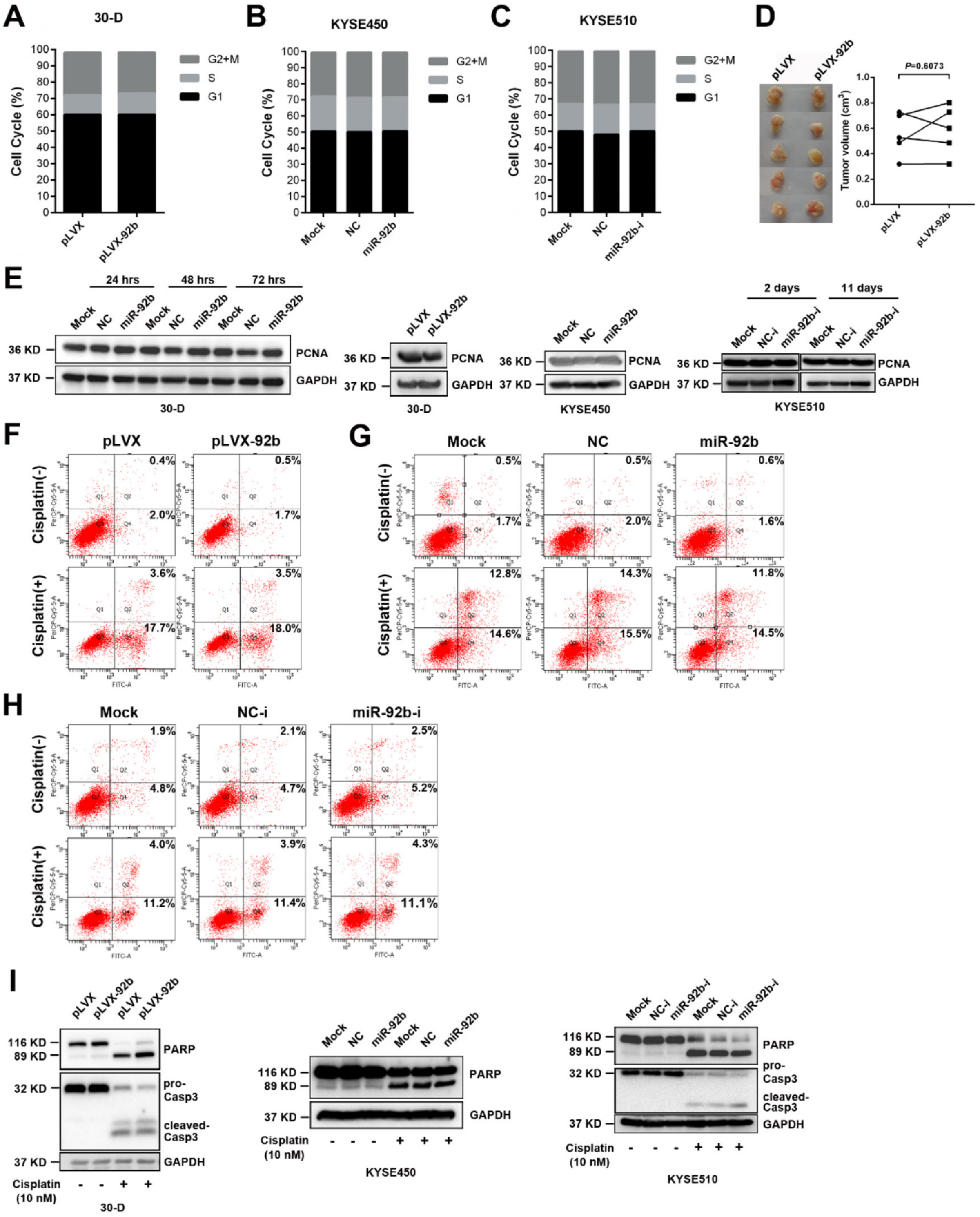

Supplementary Figure S4: MiR-92b had little influence on proliferation and apoptosis of ESCC cells. A–D. Forced expression of miR-92b did not affect cell cycle progression of 30-D (A) and KYSE450 cells (B) as shown by flow cytometry results. Decreased miR-92b (miR-92b-i) in KYSE510 cells failed to alter cell cycle progression (C). One representative pair of the control tumor and the tumor stably expressing miR-92b obtained one month after subcutaneous injection further excluded the possibility of miR-92b affecting proliferation of ESCC cells (n = 5, (D). **E.** Increased or decreased expression of miR-92b in 30-D, KYSE450, and KYSE510 cells did not change the level of PCNA. **F–H.** Flow cytometry results showed that under adherent condition, manipulation of miR-92b expression in 30-D (F), KYSE450 (G), and KYSE510 (H) cells did not affect apoptosis of these transfected cells. Cisplatin treated ESCC cells acted as positive control. **I.** Immunoblots results showed that manipulation of miR-92b expression in ESCC cells did not affect cleavage of PARP and Caspase3.

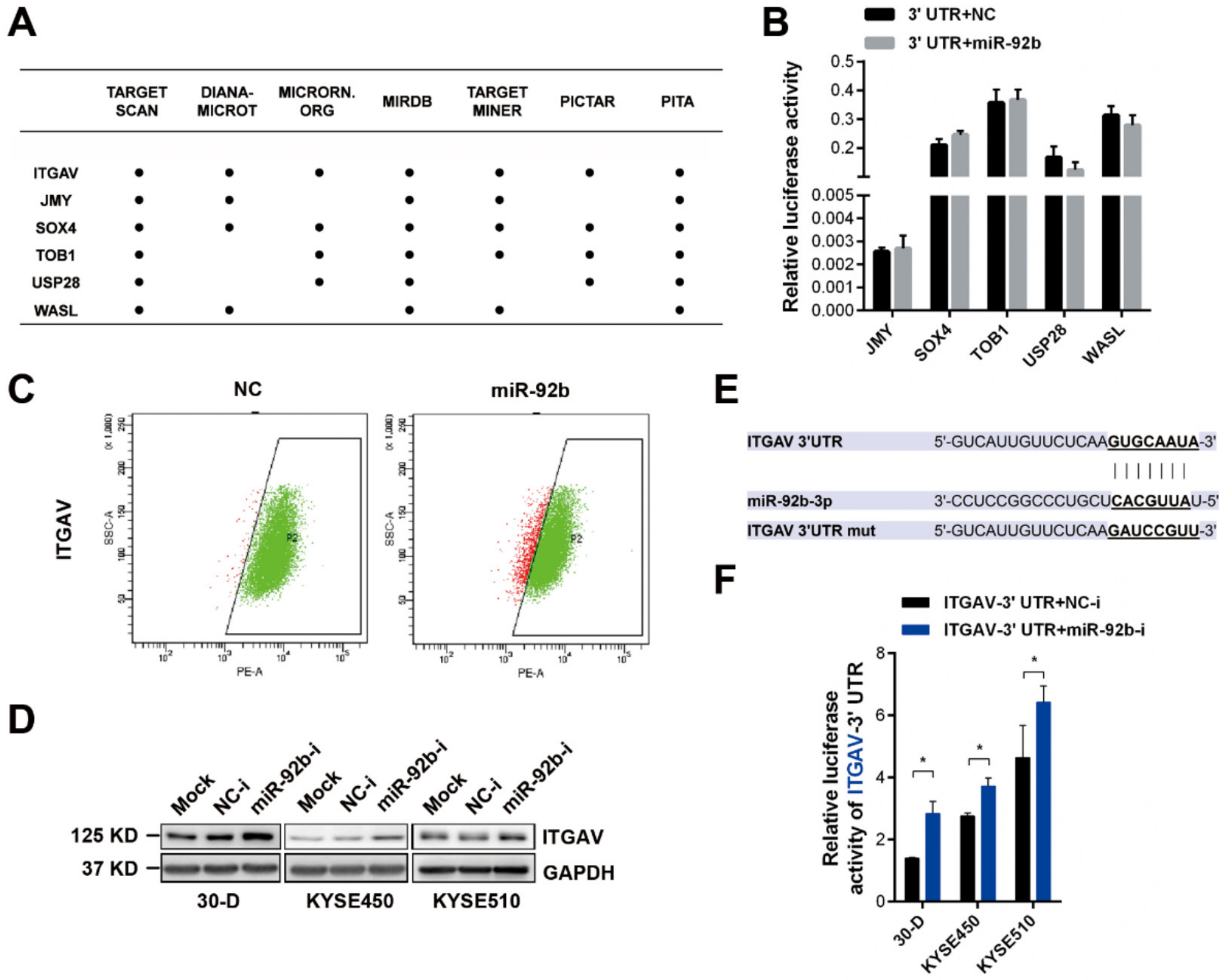

Supplementary Figure S5: ITGAV and ITGA6 were verified as targets of miR-92b. A. Six candidate targets of miR-92b were picked out using seven online algorithms. Dots in the table mean that the indicated gene was predicted as putative target of miR-92b. **B.** Luciferase reporter assay excluded the possibility of five genes (JMY, USP28, SOX4, TOB1, and WASL) as targets of miR-92b. The predicted binding sites from 3’UTR of the five genes were constructed into pISO and these plasmids were then transfected with miR-92b or control oligos (NC) respectively into 30-D cells. After 24 hr, the luciferase activity was examined. **C.** MiR-92b reduced membrane expression of ITGAV in 30-D cells as shown in flow cytometry results. Suspended one million 30-D cells transfected with control oligos and miR-92b mimic were incubated with PE- conjugated antibodies for 30 min in dark before subjected to analysis. **D.** Inhibition of miR-92b in ESCC cells led to increased level of ITGAV at protein level. **E.** Illustration of wild type and mutated binding sites of miR-92b located in 3’UTR of ITGAV. Wild type and mutated binding sites and the seed sequence of miR-92b are presented as bold capital letters. **F.** Inhibition of endogenous miR-92b enhanced luciferase activity relative to control counterparts. The luciferase activity was detected 24 hr after synthesized miR-92b inhibitor (100 nM) or control oligos (100 nM) were co-transfected with pISO plasmid containing wild type binding site of ITAGV (ITGAV-3’UTR).

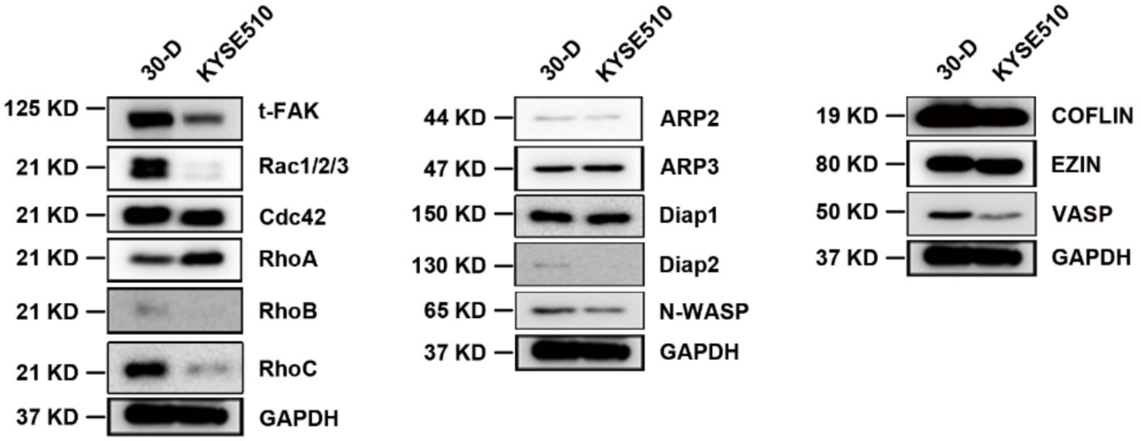

Supplementary Figure S6: Proteins essential for cellular motility were compared between 30-D and KYSE510 cells using immunoblots. In brief, higher level of FAK, Rac1/2/3, RhoC, N-WASP, and VASP were detected in 30-D cells.

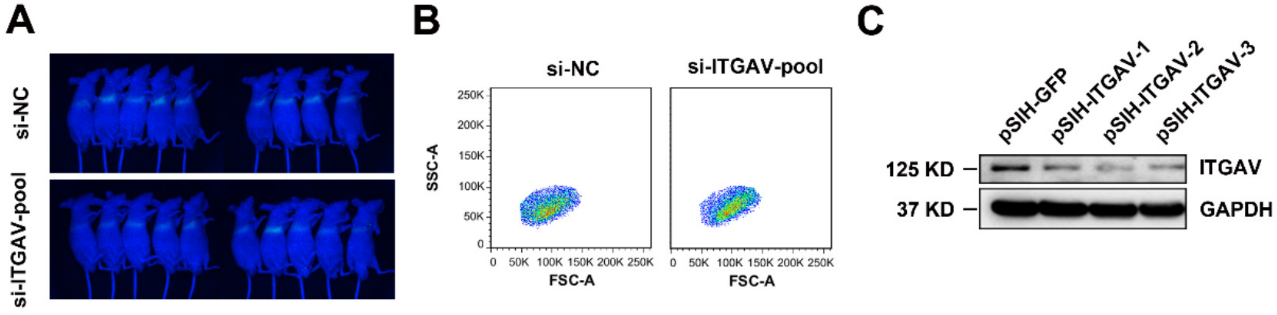

Supplementary Figure S7: ITGAV was involved in lung arrest of ESCC cells *in vivo*. A. Reduced ITGAV attenuated adhesion of 30-D cells to microvasculature in lungs. *In vivo* detections were performed less than 24 hr after introduction of cells via tail veins into the indicated number of immunocompromised mice. **B.** ITGAV reduction did not alter 30-D cells size based on forward scatter (FSC). The flow cytometry analysis was performed 48 hr after transfections of siRNAs. **C.** Stable clones from 30-D cells with reduced ITGAV were obtained by puromycin selection and the decreased level of ITGAV was examined using immunoblots.

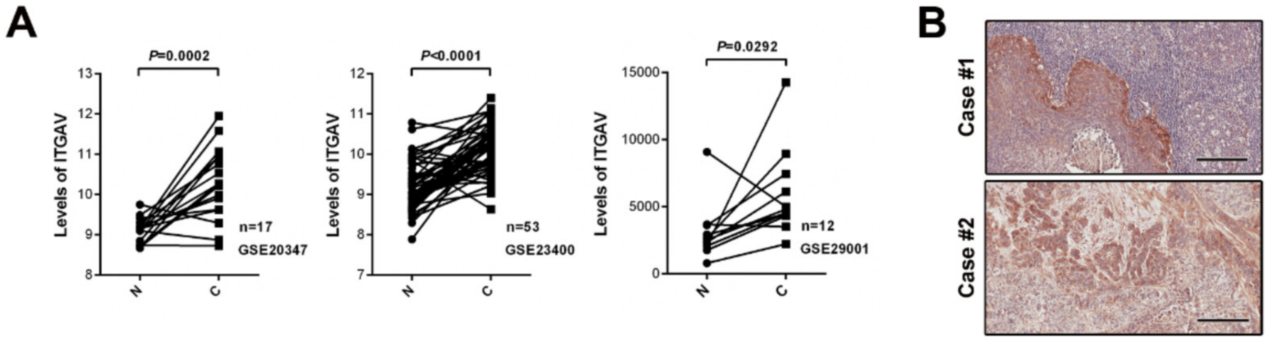

Supplementary Figure S8: Analysis of ITGAV expression in ESCC specimens. A. Three data sets of ESCC specimens from Gene Expression Ominous were analyzed, showing that ITGAV had higher expression in cancerous tissues (C) relative to adjacent normal counterparts (N). Paired Student's *t*-test was used to evaluate statistical significance. **B.** Images of IHC results show higher expression of ITGAV in the invasive fronts of ESCC specimens.

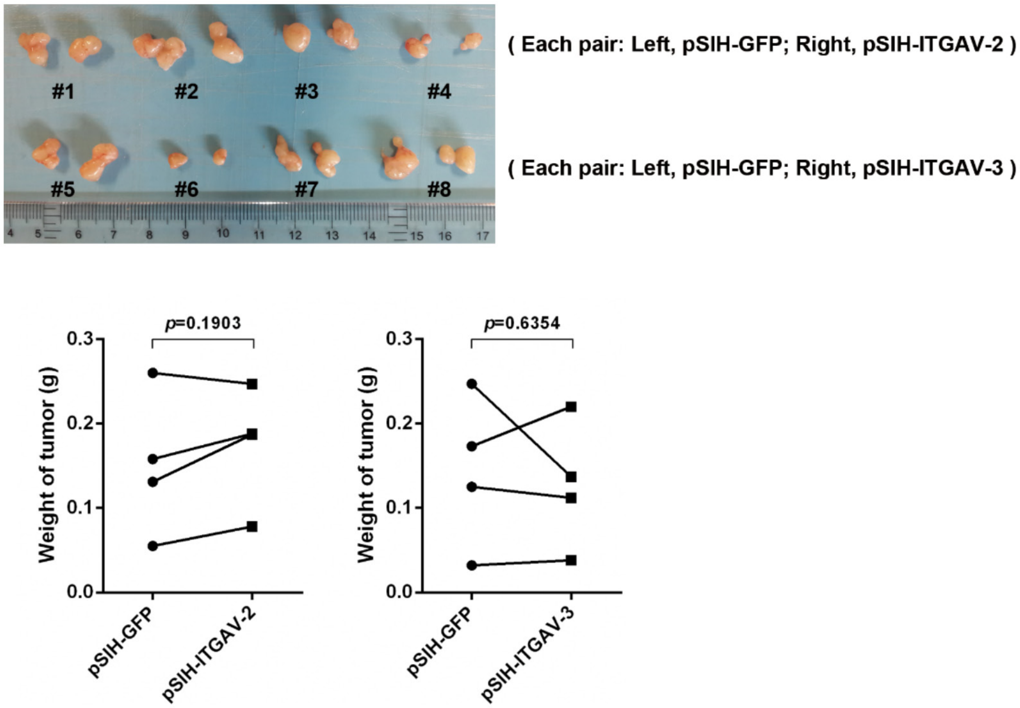

Supplementary Figure S9: ITGAV did not influence growth of ESCC cells *in vivo*. Two stable ITGAV knockdown clones were tested and significant growth alterations relative to the control cells were not detected (n = 4 for each oligos).

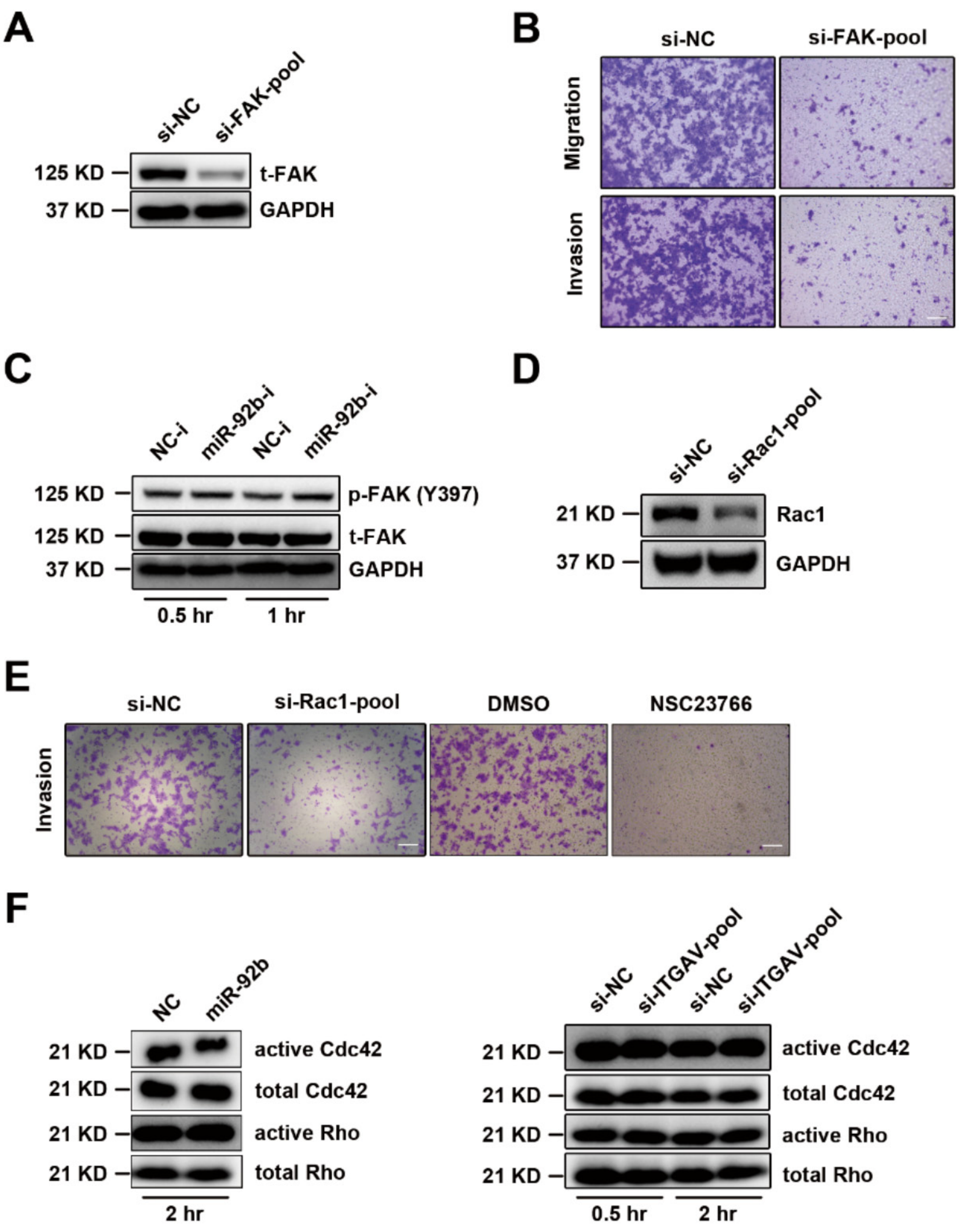

Supplementary Figure S10: FAK-Rac1 pathway was critical in modulating motility of ESCC cells. A. ESCC 30-D cells were transfected with three siRNAs in combination (100 nM) for 36-48 hr and total FAK level was detected using immunoblots. **B.** Photographs of *in vitro* migration and invasion results showed that reduced FAK caused less 30-D cells to penetrate membrane. **C.** Inhibition of endogenous miR-92b in KYSE510 cells promoted p-FAK (Y397) level without affecting total FAK (t-FAK). KYSE510 cells were transfected with miR-92b inhibitor for 96 hr and cells were harvested under chemotaxis condition at indicated time points. **D.** Knockdown of Rac1 using a pool of three siRNAs (100 nM) against Rac1 was tested 48 hr after transfection. **E.** The penetrated 30-D cells with decreased Rac1 were fewer than control counterparts as shown in migration assay *in vitro*. NSC23766, which is an inhibitor of Rac1 activation, was incubated with 30-D cells during transwell assay and suppressed motility of the treated cells relative to control cells. **F.** Increased miR-92b or decreased ITGAV in 30-D cells exerted little influence on Cdc42 and Rho activation. Control and transfected 30-D cells were harvested under chemotaxis condition at indicated time points. Scale bars, 400 μm.

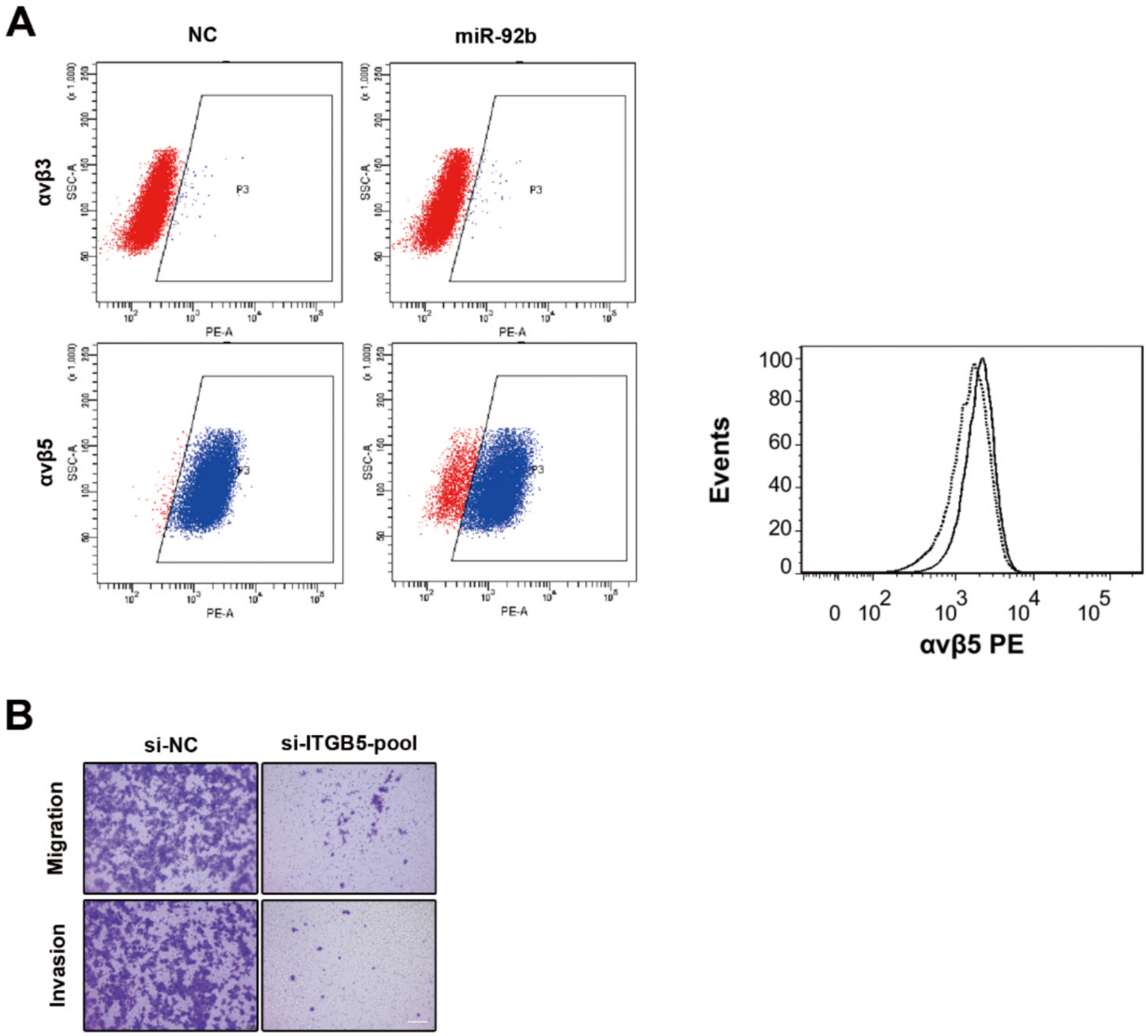

Supplementary Figure S11: Integrin αvβ5 promoted motility of ESCC cells *in vitro*. A. Membrane expression of integrin αvβ5 decreased as the result of transfection of miR-92b. Integrin αvβ3 expression was not detected in 30-D cells. Cells were incubated with PE-conjugated antibody against integrin αvβ3 or αvβ5 before they were subjected to flow cytometry analysis. **B.** Knockdown of ITGB5 using specific siRNAs against this integrin inhibits migration and invasion of 30-D cells *in vitro*. Representative photographs of transwell assay demonstrated that far fewer 30-D cells with reduced level of ITGB5 penetrated membrane than control cells did. Scale bars, 400 μm.
